# Characteristics and trends of pediatric firepit burns: insights for prevention and safety

**DOI:** 10.1136/wjps-2023-000700

**Published:** 2024-01-30

**Authors:** Maria Fazal, Charbel Chidiac, Raheel Ahmad, Oussama Issa, Erica Hodgman, Alejandro V Garcia

**Affiliations:** 1 Johns Hopkins School of Medicine, Baltimore, Maryland, USA; 2 Department of Surgery, Division of Pediatric Surgery, Johns Hopkins School of Medicine, Baltimore, Maryland, USA; 3 Division of the Biological Sciences, University of Chicago, Chicago, Illinois, USA; 4 School of Medicine, American University of Beirut, Beirut, Lebanon

**Keywords:** Preventive Medicine, Child Health

## Abstract

**Introduction:**

As fire pits grow in popularity, so do the associated burn injuries. Our study examines pediatric fire pit burns characteristics to raise awareness and promote safety precautions.

**Methods:**

We conducted a retrospective review of pediatric patients (≤21 years) with firepit burns at a tertiary care hospital from 2016 to 2021.

**Results:**

Eighty-four patients were identified, of whom 70.2% were male, with a median age of 62 months. The median percent total body surface area burned was 2% (interquartile range (IQR)=1–4). Thirty-five (41.7%) patients were admitted and 7 (8.3%) underwent grafting. Neck and trunk burns had the highest grafting rates (66% and 33%, respectively). The hands (41.7%) and the lower extremities (27.4%) were the most frequently burned body areas. The leading causes of burns were ashes/hot coals (34.5%), flames (31.0%), and direct contact (25.0%), often resulting from falling into the fire (59.5%) or running or playing in activities near it (26.2%). Thirty-five (41.7%) were admitted for inpatient management, while 49 (58.3%) were treated as outpatient. Eleven (13.2%) underwent at least one reconstructive surgery, 7 (8.4%) had at least one rehabilitation visit, and 65 (77.4%) had follow-up clinic visits. The median length of stay was 2 days (IQR=1.0–3.5). The peak months for burns were from August through October (*n*=40, 46.0%), with an increase observed from 10 cases in 2016 to 20 cases in 2020.

**Conclusions:**

Given the significant proportion of firepit burns resulting from unsafe fire behaviors, it is crucial that caretakers are aware of proper firepit safety precautions.

**Level of evidence:**

III.

**WHAT IS ALREADY KNOWN ON THIS TOPIC**

- Fire pits surged in popularity in the USA during the COVID-19 pandemic, leading to an increase in related injuries, especially among young children.

**WHAT THIS STUDY ADDS**

- Our study shows a concerning increase in pediatric burns from fire pits, most commonly in young boys under 5.
- While some injuries might be mild, others can be severe, leading to hospitalization, grafting, rehabilitation, and subsequent clinic visits.

**HOW THIS STUDY MIGHT AFFECT RESEARCH, PRACTICE OR POLICY**

- This emphasizes the need for targeted awareness campaigns and enhanced safety measures.

## Introduction

Fire pits have recently risen in popularity in backyards across the USA and are the most popular outdoor design element in the country.^1^ Fire pits come in many varieties and can be as simple as a dugout hole in the ground or an elaborate table apparatus. The sales of fire pits have seen a substantial increase since the onset of the COVID-19 pandemic, with many stores reporting a sales growth of over 300%.^2^ However, as their popularity has risen, so has the number of related injuries, with emergency department visits for firepit or outdoor heater incidents nearly tripling between 2008 and 2017.^3^ Among those affected by firepit injuries, young boys under the age of 5 are particularly susceptible, often experiencing harm from hot coals and ashes left from previous day’s campfires.^4–7^

Our study aims to describe our institutional experience with pediatric burns caused by fire pits and their trend over the years in order to determine the circumstances of these injuries and the need to raise awareness and promote safety precautions.

## Methods

We conducted a retrospective review of patients under 21 years of age presenting with firepit burns from July 2016 to June 2021 using data from a tertiary academic medical center’s pediatric trauma and burn program database. This database is prospectively maintained by trained registrars as part of the hospital’s designation as the only pediatric burn center in the state of Maryland. Information collected from patients included demographics, medical comorbidities, percent total body surface area (% TBSA) burned, area(s) burned, burn severity, injury etiology and narrative, location of incident, admission date, days of hospitalization, number of outpatient care visits, rehabilitation visits, and reconstructive operations. Additionally, grafted patients were compared with those who were not.

Continuous variables are presented as median and interquartile range (IQR) and categorical variables as frequencies and percentages. Comparison between continuous variables was performed using the Wilcoxon rank-sum test, while comparison between categorical variables was performed using the Fisher’s exact test or χ^2^ test. A p value of <0.05 was considered significant. All analyses were conducted on R V.4.2.2 (“innocent and trusting”).

## Results

Over the 5-year period, 84 patients in total presented to our institution with burns caused by fire pits. Most of the patients were boys (*n*=59, 70.2%) aged 1–5 years old (*n*=48, 57.1%), with a median age of 62 months. The vast majority of patients were Caucasian (*n*=68, 81.0%), with other races including black or African American (*n*=5, 6.0%), Asian (*n*=2, 2.4%), American Indian/Alaskan Native (*n*=1, 1.2%), and other (*n*=7, 8.3%). Hispanic patients made up 11.9% (*n*=10) of the cohort. Fifteen patients (17.9%) had comorbidities, the most common being asthma (*n*=4, 4.8%). Psychiatric comorbidities such as autism spectrum disorder, attention deficit hyperactivity disorder, obsessive compulsive disorder, and anxiety were present in 4.8% (*n*=4) of the patients. Patient and burn characteristics are further broken down in table 1.

**Table 1:** Demographic and burn characteristics of patients presenting with burns caused by fire pits

	Overall (*N*=84)	Grafted		*P* value
	*n* (%)	Yes (*n*=7) *n* (%)	No (*n*=77) *n* (%)	
Age (months), median (IQR)	62 (37–116.5)	72 (49–89)	61 (37–121)	0.73
Female	25 (29.8)	2 (28.6)	23 (29.9)	1.00
Race				0.58
Caucasian	69 (82.1)	6 (85.7)	63 (81.8)	
Black or African American	5 (6.0)	1 (14.3)	4 (5.2)	
Asian	2 (2.4)	0 (0)	2 (2.6)	
American Indian/Alaskan Native	1 (1.2)	0 (0)	1 (1.3)	
Other race	7 (8.3)	0 (0)	7 (9.1)	
%TBSA, median (IQR)	2 (1–4)	7 (4.5–7.7)	1.65 (1.0–3.0)	**<0.0001**
Body region affected*				0.23
Head	17 (20.2)	2 (28.6)	15 (19.5)	
Neck	3 (3.6)	2 (28.6)	1 (1.3)	
Trunk	12 (14.3)	4 (57.1)	8 (10.4)	
Upper extremities	21 (25.0)	5 (71.4)	16 (20.8)	
Hand(s)	35 (41.7)	5 (71.4)	30 (39.0)	
Buttock	4 (4.8)	1 (14.3)	3 (3.9)	
Genital area	2 (2.4)	0 (0)	2 (2.6)	
Lower extremities	23 (27.4)	4 (57.1)	19 (24.7)	
Feet	10 (11.9)	0 (0)	10 (13.0)	
Burn source				0.46
Campsite	15 (17.9)	1 (14.3)	14 (18.2)	
Garden or yard	40 (47.6)	4 (57.1)	36 (46.8)	
Beach	3 (3.6)	1 (14.3)	2 (2.6)	
Religious institution	1 (1.2)	0 (0)	1 (1.3)	
Restaurant or cafe	1 (1.2)	0 (0)	1 (1.3)	
Unspecified	24 (28.6)	1 (14.3)	23 (29.9)	
Burn type				0.58
Ashes	29 (34.5)	2 (28.6)	27 (35.1)	
Flame	26 (31.0)	4 (57.1)	22 (28.6)	
Flash	8 (9.5)	0 (0)	8 (10.4)	
Contact	21 (25.0)	1 (14.3)	20 (26.0)	
Burn degree*				**<0.0001**
First degree	7 (8.3)	0 (0)	7 (9.6)	
Second degree	78 (92.9)	6 (85.7)	72 (98.6)	
Third degree	15 (17.9)	7 (100)	8 (11.0)	
Length of stay (days), median (IQR)	2 (1.0–3.5)	8 (7.0–10.0)	1.5 (1.0–3)	**<0.0001**
Follow-up clinics visits	65 (77.4)	6 (85.7)	59 (76.6)	<0.0001
Number of visits, median (IQR)	2 (1.0–3.0)	3 (1.5–5.0)	2 (1.0–2.5)	
Rehabilitation visits	7 (8.4)	2 (28.6)	5 (6.5)	**0.02**
Number of visits, median (IQR)†	1 (1.0–3.0)	2.5 (1.0–4.0)	1 (1.0–2.5)	
Reconstructive operations	11 (13.2)	7 (100)	4 (5.2)	**0.02**
Number of operations, median (IQR)†	2 (1.0–6.0)	2 (1.0–5.0)	3.5 (1.0–9.0)	

Values in bold signify significance of p <0.05.

*Percentages add up to more than 100% as one patient can have burns in more than one body area.

†Only patients who had a rehabilitation visit or a reconstructive operation were included in the calculation of the median number.

IQR, interquartile range; %TBSA, percent total body surface area.

Of the 84 patients, 35 (41.7%) were admitted for inpatient management, while 49 (58.3%) were treated as outpatient. Admitted patients had a median length of hospitalization of 2 days (IQR=1–3.5). The median %TBSA burned was 2% (IQR=1–4), with 39 (46.4%) patients having a TBSA burned between 1% and 3%. Only three patients (3.6%) had a TBSA burned greater than 10%. The majority of patients (*n*=78, 92.9%) had at least one second-degree burn and 15 patients (17.9%) had third-degree burns. Sixty-five patients (77.4%) experienced a partial thickness burn (second-degree burn) as the highest burn severity. Fifty-six patients (66.6%) underwent debridement and seven patients (8.3%) graft placement. Patients who underwent graft placement had a greater %TBSA (7% vs 1.5%, *p*<0.0001) and more third-degree burns (100% vs 11.0%, *p*<0.0001) compared with those who did not.

Out of all the patients, 11 (13.2%) underwent at least one reconstructive surgery with a median of 2 surgeries (IQR=1.0–6.0), 7 (8.4%) had at least one rehabilitation visit with a median of 1 visit (IQR=1.0–3.0), and 65 (77.4%) had follow-up clinic visits related to the burn with a median of 1 visit (IQR=1.0–3.0). Grafted patients had more reconstructive surgery (100% grafted vs 5.2% non-grafted, *p*=0.02), rehabilitation session (28.6% vs 6.5%, *p*=0.02), and follow-up clinic visits (median 3 vs 2 visits, *p*<0.001) than non-grafted patients (table 1).

**Figure 1:**
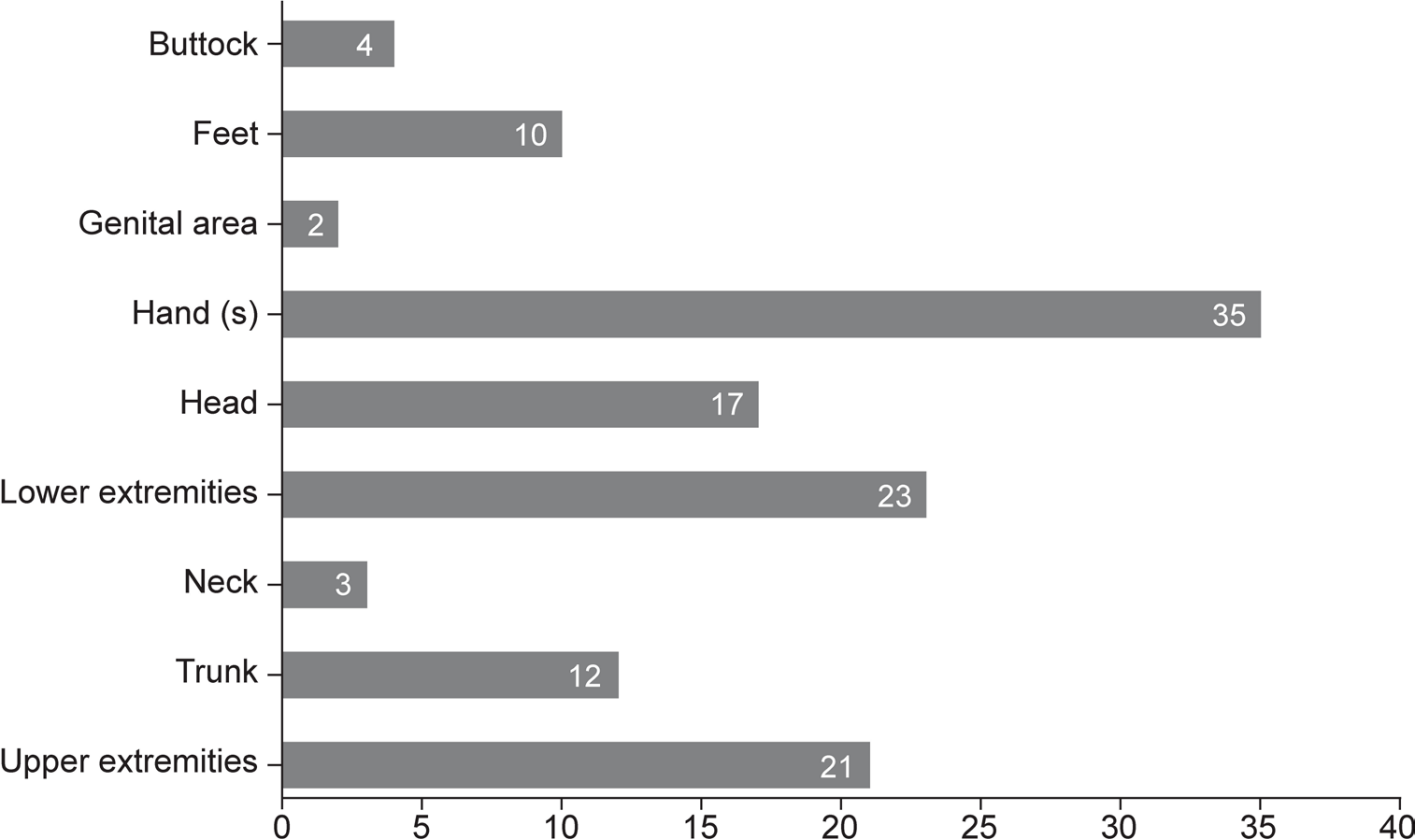
Number of burns by body region affected.

The most common body areas burned were the hands (*n*=35, 41.7%), lower extremities (*n*=23, 27.4%), upper extremities (*n*=21, 25%), and head (*n*=17, 20.2%). It is important to note that a single patient can have burns in multiple body areas, which were recorded separately. Other body regions were also affected by burns, as shown in figure 1. Among the patients who were grafted, the body areas involved were the head (*n*=2), neck (*n*=2), trunk (*n*=4), upper extremities (*n*=5), hands (*n*=5), buttocks (*n*=1), and lower extremities (*n*=4).

In terms of the setting where firepit burns occurred, approximately half of the incidents (*n*=40, 47.6%) took place in private residences’ gardens or yards. Other locations included campsites (*n*=15, 17.9%), beaches (*n*=3, 3.6%), a restaurant (*n*=1, 1.2%), and a religious institution (*n*=1, 1.2%). The primary causes of the burns were ashes/hot coals (*n*=29, 34.5%) and flames (*n*=26, 31.0%). Among the burns caused by ashes/hot coals, a significant portion (*n*=10, 35.7%) were from fire pits that were lit the previous day. Contact burns accounted for a quarter of the injuries (*n*=21, 25.0%). The primary cause of these contact burns was coming into contact with a metal structure (*n*=10, 47.6%) of the fire pit, such as a lid or grate. Other contact burns were caused by direct contact with various hot or ignited objects, including clothing, plastic, wood, stones, and food. The majority of the children were burned after falling into the fire (*n*=50, 59.5%), while several incidents occurred as a result of running or playing near the fire (*n*=22, 26.2%). Other activities preceding burns included the addition of accelerants to the fire (*n*=9, 10.7%), cooking food over the fire (*n*=4, 4.8%), and the explosion of an oil or lava lamp placed close to the fire (*n*=2, 2.4%).

**Figure 2:**
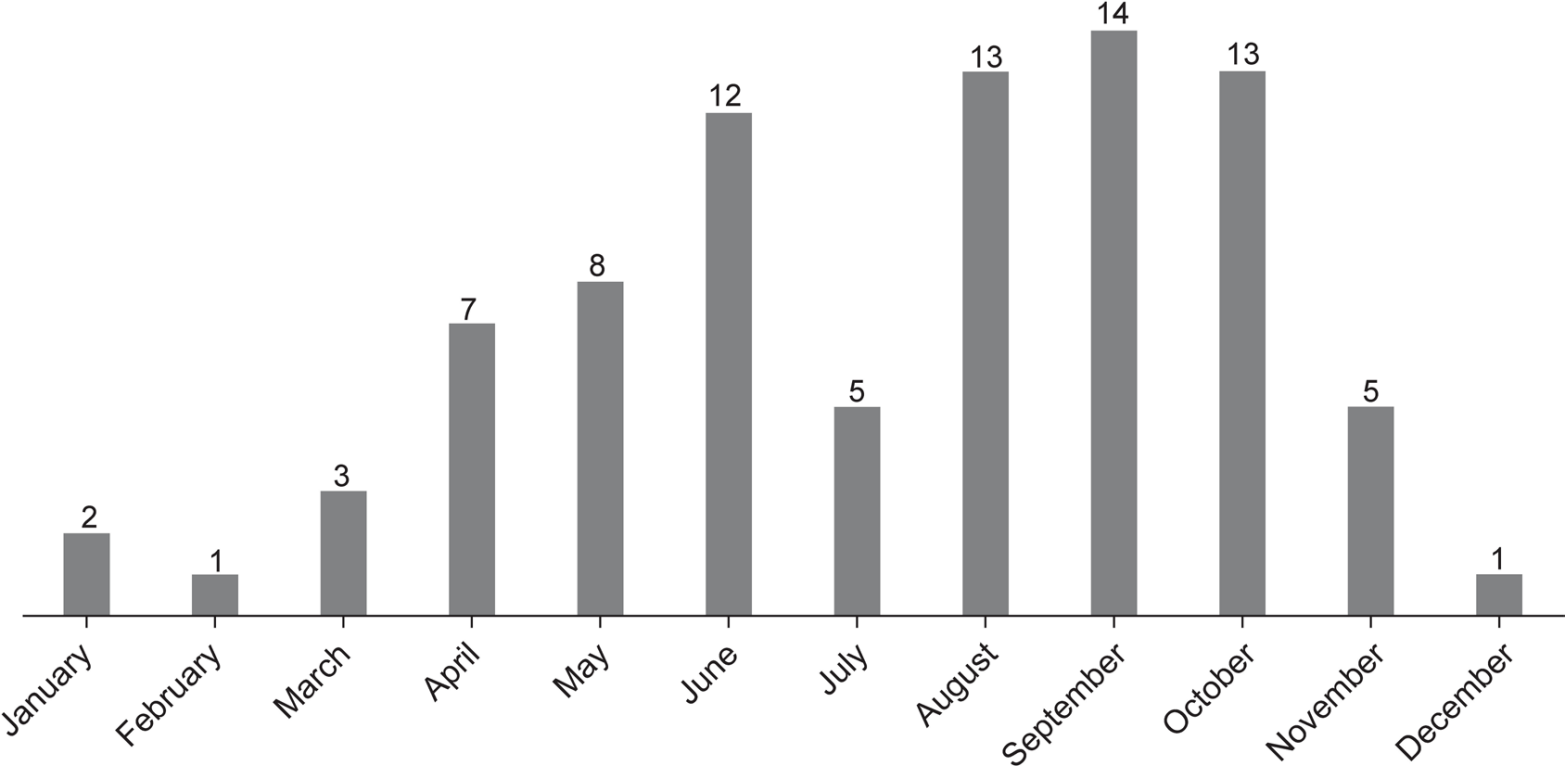
Number of children who presented each month with firepit burns.

**Figure 3:**
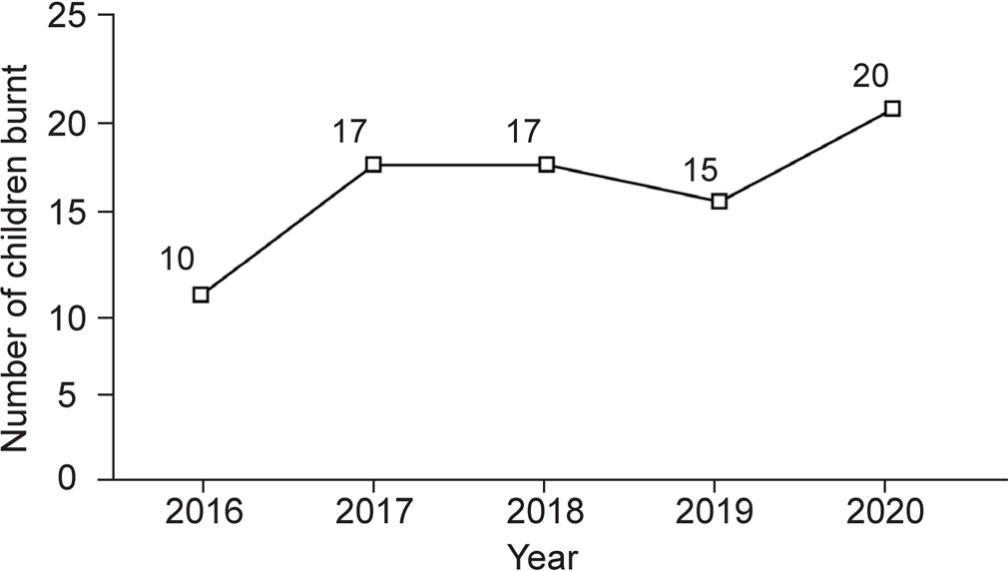
Number of children who presented each year from 2016 to 2020 with firepit burns.

In terms of seasonal distribution, nearly half of the burns (*n*=40, 46.0%) occurred between August and October (figure 2). The number of yearly burns showed an increasing trend from 2016 (*n*=10) to 2017 (*n*=17), remained stable in 2018 (*n*=17), decreased in 2019 (*n*=15), and reached the highest number in 2020 (*n*=20) (figure 3).

## Discussion

Due to the rising popularity of fire pits in the USA, burns caused by them are increasing, especially in the pediatric population. Our study describes our institutional experience with pediatric burns caused by fire pits. We found that the population most affected was young boys under the age of 5 years. Injuries occurred mostly due to falls and running or playing near a fire pit and most commonly led to hand burns. In addition, we found an increase of cases from 2016 to 2020, with peak months for burns being August, September, and October. This emphasizes the importance of awareness about fire pits and their safety precautions during the fall months, especially among caregivers.

Previous literature on outdoor recreational fires found that burns were most common in male children under 5 years of age and occurred most frequently on the hands, similar to our findings.^4–7^ Additionally, similar to other studies, we found that most of the burn injuries related to fire pits were due to falls and that the most commonly injured body part was the hand.^5–7^ Contrary to a national review of firepit injuries by Flaherty and Sheridan,^7^ which found that only 10% of injuries were due to hot ashes and/or coals, a third of the patients in our cohort were burned due to hot ashes and/or coals.

In our study, we found that over a quarter (26%) of the burns were due to unsafe behaviors, such as the child running or playing near a fire. This highlights a need to continue raising awareness of proper fire safety behaviors. Because young children have little understanding of fire dangers, and poor dexterity and coordination compared with adults, strategies related to child fire safety are centered around maintaining a safe distance from fire. A popular safety strategy for outdoor fires is drawing or tracing a “circle of safety” to prevent children from coming within 4 feet of a fire.^8^ For very young children, a child safety fence may be used instead. Strategies to prevent fire play in young children include assigning dangerous objects, such as matches, fuel, and the firepit structure itself, as “adult objects” that are not to be touched.^9^ Most of the patients were injured at a private residence garden or yard, possibly due to decreased precautions in a comfortable home setting. None of the children under 10 were unsupervised, highlighting that serious fire injuries can happen in an instant.

Importantly, the majority of patients required follow-up clinic visits, while some required additional rehabilitation sessions and reconstructive operations. This shows that firepit burns place a burden on the individuals, their families, and the healthcare system, highlighting the importance of raising awareness on firepit precautions.

Because one-third of injuries were due to hot ashes/coals, and a significant proportion of these were due to a day-old fire, it is important to emphasize the importance of extinguishing these fires fully with water rather than sand and to create a temporary barrier around the extinguished fire pit. Families should also be reminded to maintain the “zone of safety” around the fire pit all the time, not just when the fire is lit. Additionally, since several of the contact burns were due to touching a metal portion of the firepit structure, injuries could be reduced by manufacturers modifying the design of store-bought fire pits. With changes in both caregiver education and structural design, there can be improvements in future firepit burn morbidity and mortality in children.

It is important to acknowledge the limitations of our study. While our center serves as the only dedicated pediatric burn center in our state, we recognize that our findings may not fully represent all fire pit-related injuries that occurred state-wide. Some individuals may have sought care at local emergency departments and were not included in our study, while others with minor injuries may not have sought medical attention at all. Another limitation is the relatively small sample size and the relatively short study period. These factors may restrict the generalizability of our findings. Further research using nationally representative samples would provide a more comprehensive understanding of temporal trends and risk factors related to firepit injuries.

In conclusion, our study provides valuable insights into pediatric firepit burns, emphasizing the importance of fire safety awareness and prevention strategies. The increase in burns, primarily due to unsafe behavior, highlights the importance of maintaining a safe distance and modifying firepit design. By educating caregivers, enhancing structures, and raising awareness, we can reduce the incidence of these injuries and protect children’s well-being. Further research and evaluation of prevention efforts are warranted for comprehensive injury reduction.

## Data Availability

No data are available.
